# Fistule urogénitale à Sikasso: à propos de 150 cas

**DOI:** 10.11604/pamj.2019.33.133.16455

**Published:** 2019-06-21

**Authors:** Salifou Issiaka Traore, Ousmane Dembele, Soumaila Traore, Aly Diallo, Amadou Maiga, Malla Sylla, Adan Maiga, Moussa Kante

**Affiliations:** 1 Service Urologie, Hôpital de Sikasso, Sikasso Ville, Mali

**Keywords:** Fistule urogénitale, obstruction du travail, la classification anatomopathologique, Urogenital fistula, obstructed labour, anatomopathologic classification

## Abstract

L'objectif de cette étude était d'analyser les aspects épidémiocliniques, thérapeutiques et anatomopathologiques de la fistule urogénitale (FUG). L'étude rétrospective porte sur les cas de FUG admis dans le Service de Chirurgie Générale et Gynéco-obstétrique entre le 1^er^ janvier 2014 et le 30 décembre 2015 y compris les 5 premières campagnes du projet fistule-Mali. La FUG occupe 19,53% de nos activités urologiques. L'âge moyen au 1^er^ mariage: 16,57 ans. La majorité (96,70%) de nos patientes étaient analphabètes et non salariées provenant à 85,36% des communes rurales. La stagnation du travail était l'étiologie dominante avec 91,50% de mort-né. Les patientes étaient primipares à 43,33% et, parmi elles 53,60% n'ont effectué aucune consultation prénatale. Le taux de divorce lié à la maladie était estimé à 7,30%. La plupart des patientes ont bénéficié d'une fistulorraphie simple dont 121 par voie basse, 26 par voies hautes et 3 par la voie mixte. Les résultats ont été satisfaisants chez 65,33% et mauvais chez 34,66% des patientes. Les fistules type I et type V ont montré les plus forts taux de succès comparés aux fistules types IV. La fistule urogénitale demeure un réel problème de santé publique. Le traitement est surtout chirurgical et son pronostic est compromis par l'étroitesse du champ, la complexité des lésions et l'état du tissu environnant. L'accent doit être mis sur la promotion socioéconomique des filles et l'accessibilité aux soins obstétricaux d'urgence. La recherche et les échanges doivent continuer afin de faciliter la mise au point d'une classification standard.

## Abstract (en)

This study aims to analyze the epidemiological, clinical, therapeutic and anatomopathologic features of urogenital fistulas. We conducted a retrospective study of patients with urogenital fistulas admitted to the department of general surgery and gynecology and obstetrics between January 1, 2014 and December 30, 2015, including the first 5 fistula repair campaigns organized by the Fistula Mali project. Urogenital fistulas account for 19.53% of all urologic disorder treated during our daily practice. The median age at first marriage was 16.57 years. The majority of our patients (96.70%) was illiterate and self-employed, coming from rural areas (85.36%). Obstructed labour was the dominant etiology, with 91.50% of stillborn children. The patients were primiparous women (43.33%) and, among them, 53.60% received no prenatal care. The divorce rate associated with the disease was 7.30%. Most patients underwent simple fistulorraphy, of whom 121 underwent lower fistulorraphy, 26 upper fistulorraphy and 3 mixed fistulorraphy. Outcomes were satisfactory in 65.33% patients and poor in 34.66% of patients. Patients with type I and type V obstetric fistulas had the highest healing rate compared to patients with type IV fistulas. Urogenital fistulas are a real public health problem. Treatment is mainly based on surgery and prognosis is compromised by the narrowness of the surgical field, the complexity of the lesions and the condition of the surrounding tissue. The focus must be on the implementation of women’s socio economic development programs and on emergency obstetric care access. The research and discussion should continue to facilitate the development of a standard classification.

## Introduction

La fistule urogénitale (FUG) est une communication anormale acquise entre les voies urinaires et génitales. Contrairement aux pays occidentaux, elle est surtout d'origine obstétricale dans nos pays sous-développés faisant suite à un accouchement dystocique et souvent associé à une fistule recto vaginale. Selon l'Organisation Mondiale de la Santé, chaque année 50000-100000 femmes développent la fistule obstétricale dont la majeure partie se trouve en Afrique subsaharienne avec une incidence de 1,24/1000 naissances [1,2]. La FUG est une pathologie grave à cause de la souffrance physique qu'endurent les patientes et, des conséquences psychosociales et économiques néfastes qu'elle engendre. Parallèlement à l'évolution de la chirurgie de la fistule, dans un souci de collaboration et de comparaison des résultats, nous avons assisté durant ces cinquante dernières années à une multiplication des systèmes de classification pronostique. Mais, force est de reconnaitre qu'aucune d'entre elles ne fait l'unanimité. Les objectifs de cette étude étaient: 1) analyser les aspects épidémiologiques, sociodémographiques et étiologiques de la FUG; 2) décrire les aspects anatomopathologiques et évaluer la PEC thérapeutique de la FUG; 3) évaluer le système de classification du CHU-Point G.

## Méthodes

Il s'agit d'une étude transversale rétrospective réalisée dans le Service de Chirurgie Générale et de Gynéco-obstétrique de l'Hôpital de Sikasso, allant du 1^er^ janvier 2014 au 30 décembre 2015 y compris les 5 premières campagnes du projet fistula-Mali. Les registres d'hospitalisation, de CRO et les dossiers des patientes ont été utilisés pour collecter les informations par rapport à: l'âge, la provenance, le statut matrimonial, les antécédents gynéco-obstétricaux, l'aspect anatomopathologique des fistules et la prise en charge thérapeutique des patientes. Les fistules ont été classées selon la classification CHU-Point G (Tableau 1). L'analyse des données a été effectuée par SPSS 20. Nous avons obtenu le résultat suivant: CI: 95% P ≤ 0,05.

**Tableau 1:** Caractéristiques anatomopathologiques des fistules (classification CHU-Point G)

Type de fistule	Caractéristiques anatomopathologiques	Effectif
**Type I**	Fistule punctiforme Cloison Vésico-vaginale	20
**Type II**		
	Vesico-cervico-urétrale	36
	IIA: sans destruction urétrale	32
	IIB: avec destruction urétrale	4
**Type III**	Trigono-cervico-utero-vaginale	18
**Type IV ou complexe**	Fistule large touchant les deux cols + sclérose vag /fistules mixtes	24
**Type V**		
	Fistule haute (iatrogène)	19
	Uretero-vaginale:	4
	Retro trigonale:	12
	Vesico-uterine:	3
**Fistule Recto-vaginale**	Fistule localisée entre le rectum et le vagin	3
**Fistule résiduelle**	Fistule dont le diamètre a été considérablement réduit par les tentatives précédentes	30

## Résultats

**Figure 1:**
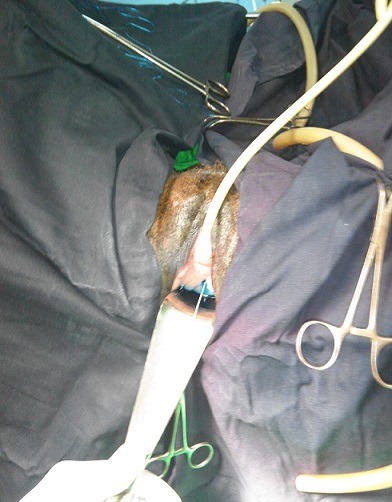
Image peropératoire JPEG d'une fistule type I cathétérisée à travers laquelle on aperçoit une fuite de bleu méthylène

**Figure 2:**
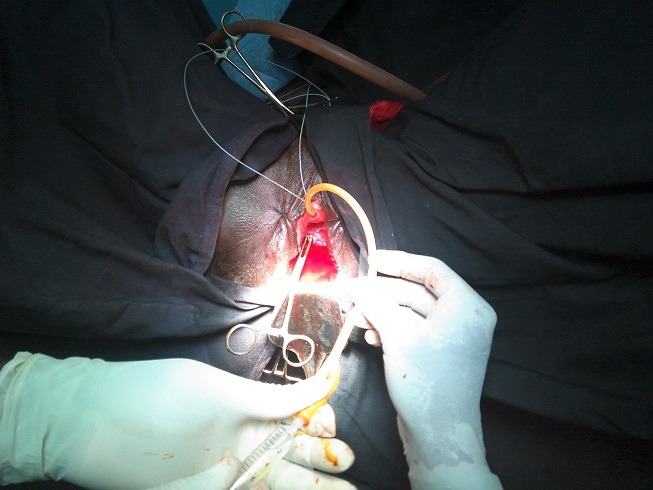
Image JPEG per opératoire de fistule type III large à travers laquelle Les méats urétéraux ont été cathétérisés

**Figure 3:**
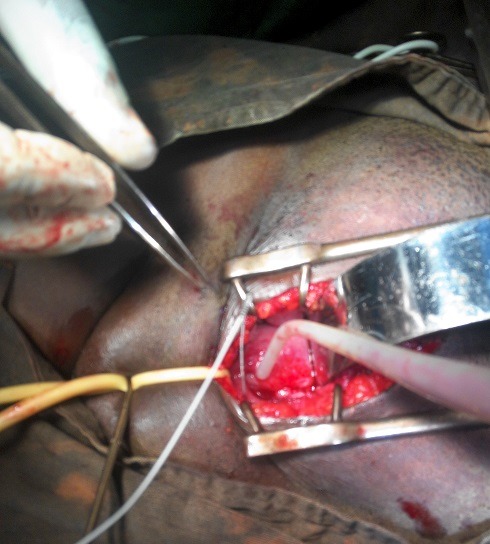
Image JPEG d'une fistule Type V catheterisée à travers cystotomie

Au total 150 patientes ont été opérées pour FUG, soit une prévalence de 19,53% sur l'ensemble de nos activités opératoires urologiques. L'âge moyen des patientes était de 35,8 ans (16-80 ans), leur moyenne d'âge au 1^er^mariage était de 16,57 ans (13-22 ans). La grande majorité était constituée de femmes mariées n'exerçant aucune activité génératrice de revenu, analphabète à 96,7% et provenait essentiellement des communes rurales loin des hôpitaux de référence. Elles étaient surtout de nationalité malienne à 62,66%, ivoirienne à 18,7% et burkinabé à 4% des cas. Quatre-vingt patientes (53,30%) n'ont effectué aucune consultation prénatale. La prédominance de l'étiologie obstétricale était évidente soit 70,53% de l'effectif suivi des fistules iatrogènes (Figure 1). La durée moyenne de travail (DMT) était de 50,36±22,66 heures. A l'issue d'accouchement dystocique, en plus d'être victime de fistule, 91,50% d'entre elles ont donné naissance à un mort-né. L'incapacité de contrôle des urines et/ou des selles a engendré de graves troubles psychosociaux et économiques qui sont entre autres: le divorce/abandon du domicile conjugal (7,30%); atrésie vaginale associée à une dyspareunie chez 14,66% des patientes, malgré que leur prise en charge (PEC) chirurgicale soit couronnée de succès. Nous enregistrons la présence de différents types de fistules, toute étiologie confondue (Figure 1, Figure 2). Le délai moyen de prise en charge (PEC) chirurgicale a été assez long (11,78 ±10,89 mois) avec une patiente qui a dû attendre 60 ans pour bénéficier d'une 1^ère^cure. La PEC chirurgicale (Tableau 2) a été possible chez 92% des patientes grâce aux soutiens matériel et financier du projet USAID/Fistula-Mali. Soixante-dix (46,66%) patientes étaient à leur première cure et 36,10% étaient à leur 31^ème^cure intervention au moins. Cette PEC a consisté soit en: une fistulorraphie simple avec dédoublement vésico-vaginal ou une réimplantation uretèro-vésicale sous anesthésie locorégionale (ALR) suivie d'un drainage urinaire par sondage urètro-vésicale pendant 14-21 jours. Le résultat de la PEC thérapeutique après 4 à 8 mois de recul est le suivant: bon chez 96 patientes soit 64,00% pouvant contenir l'urine et l'émettre au moment voulu; une amélioration chez 1,33% de nos patientes dont la fistule a été anatomiquement fermée mais perdant les urines par moment et un échec total chez 34,66% de nos patientes (Figure 3). Les fistules type I et type V ont été celles qui ont donné les plus forts taux de succès avec respectivement 95% et 80%. Les taux d'échec et de complication les plus élevés ont été enregistrés avec les fistules type IV (P < 0,05).

**Tableau 2:** Récapitulatif des aspects liés à la prise en charge chirurgicale

Type fistule	Voie d’abord	Complication PO	Durée M
**Type I**	Voie basse 100%	Absence COP	43,50 min
**Type II**	Voie basse 100%	IU Persistante (5,50%)	56,25 min
**Type III**			
	V Basse (72%)	Hémorragie PO (11,50%)	80,50 min
	Episiotomie bilatérale (11,11%)		
	28% V haute (Risque trauma Méats urétéraux)	A/Dyspareunie: 16,66%	
**Type IV**			
	V Mixte (8,50%)	Hémorragie PO (25%)	125 min
	V basse (91,50%)	Ac Thrombo-E (4,15%)	
	Episiotomie bilatérale (25%)	Atre /Dyspareunie (41,50%)	
**Type V**	Voie haute 100%	ISO fistule vesico cut (31,50%)	75,75 mi

## Discussion

Le sentiment de honte suscité par cette pathologie dévalorisante et l'absence de système de recherche active des cas à l'échelle communautaire font que subsistent des difficultés pour évaluer l'incidence réelle de cette pathologie. Mais, sa prévalence dans notre série était assez élevée (19,53%), comparée à d'autres équipes africaines [3]. A l'instar d'autres études [4], la fistule obstétricale reste dominante dans notre série avec comme principale étiologie la nécrose ischémique secondaire à une compression prolongée du tissu mou entre la présentation et l'anneau pelvien au cours d'un accouchement dystocique. Cette prédominance a été confirmée par une DMT assez longue et la proportion assez importante (74%) de patientes dont la durée du travail a excédé les 48 heures. L'analyse des aspects socioéconomiques réconforte la littérature [5,6]; la précocité du mariage et son corollaire de grossesse précoce, l'absence d'activité génératrice de revenu, le faible niveau d'instruction ainsi que les difficultés d'accès aux soins obstétricaux d'urgence de qualité exposent à un risque de fistule obstétricale.

Concernant les conséquences psychosociales engendrées par cette pathologie, nous enregistrons un taux de divorce/abandon domicile conjugal assez bas comparé à celui d'autres auteurs africains [7], mais notre taux de mort-né est aussi élevé que dans beaucoup d'autres séries [8, 9]. Cette forte corrélation entre la principale cause qu'est la stagnation du travail et un taux de mort-né élevé dans notre série, confirmerait l'assertion selon laquelle la femme victime de fistule n'est autre qu'une rescapée de la mort [10]. Quel que soit l'expérience et la dextérité du chirurgien, la réussite d'une chirurgie de la fistule, repose sur deux éléments essentiels: 1) assurer une bonne étanchéité; 2) réunir les conditions tant générales que locales pour une bonne cicatrisation; 3) la technique de fistulorraphie simple décrite par Camey [11] a été privilégiée avec comme mode d'exposition: la traction sur sonde introduite à travers l'orifice externe et dont le ballonnet est gonflé dans la vessie; la traction sur des pinces d'alice placées autour de l'orifice externe ou le refoulement successivement des commissures postérieure et antérieure à l'aide de beniqué introduit à travers l'orifice et le méat urétral. Nous disons qu'avec un taux de succès global de 64,00% de fistule fermée avec possibilité de miction spontanée et un taux d'échec estimé à 34,66%, notre résultat est inférieur à celui d'autres auteurs [12]. Chose qui pourrait s'expliquer par diverses raisons: le retard de cicatrisation dû probablement à l'infection, la dénutrition, la mauvaise trophicité; la nature de la population liée aux types de fistule et le caractère formatif par compagnonnage des interventions.

La comparaison des trois systèmes (Tableau 3) de classification à travers l'analyse des résultats de trois séries dont la nôtre [13, 14], prouve l'existence d'une certaine convergence des points de vue concernant le pronostic. En effet, les fistules simples (type I CHU-Point G, typeI Waaldjik, type III Benchekroune) et les fistules hautes iatrogènes (typeV CHU PointG, Type III Waaldjik) ont une forte chance de succès. Tandis que les fistules complexes (types IV CHU Point G, TypeI Benchekroune) ont non seulement un mauvais pronostic thérapeutique, une durée d'intervention relativement longue mais aussi un taux élevé de complication péri-opératoire. L'inversion utérine puerperale (IUP) est surtout l'apanage des fistules uretro-cervicales (Type II Ac type II B du CHU-Point G; TypeII Waaldjik; Type II Benchekroune), quel que soit la technique chirurgicale adoptée et cela malgré un bon résultat anatomique. Cependant, il existe une divergence entre ces différentes classifications quant à l'intérêt accordé aux fistules trigono-cervico-utero-vaginale ou type III du CHU-Point G, divergence due surtout à la non identification de cette entité dans la classification de Waaldjik et au fait qu'elle n'a pas été individualisée dans la classification de Benchekroune. Malgré l'existence de nombreuses classifications [15, 16], aucune ne fait encore l'unanimité. On sait que la valeur d'une classification réside dans sa capacité de décrire avec précision les lésions, d'aider à la prise de décision par rapport au choix de la modalité thérapeutique, d'évaluer les difficultés liées à cette prise en charge en termes d'effort et de pronostic. Bien qu'elle ne soit pas parfaite, la classification CHU-Point G a pu montrer une certaine valeur dans l'évaluation du pronostic thérapeutique, le choix de la voie d'abord et l'estimation des difficultés et morbidités péri opératoire.

**Tableau 3:** Comparaison des 3 systèmes de classifications à travers 3 séries

	Type I	Type II	Type III	Type IV	Type V
**Notre série**	Succès 95%	Succès 44%	Succès 50%	Echec 84%	Succès 80%
**Class CHU PointG**		ICUP 5,55%	Hémorragie: 11,50%	Hémorragie: 25%	ISO 31,50%
			A/Dyspareunie 16,66%	A /Dyspareunie: 41,50%	
**A B Diallo**	Succès 93,33%	Succès 73%	Type III non isolé (Camoufle dans les fistules complexes)	Echec 35%	Non identifié
**Class Benchkroune**		ICUP 5,0%			
**Bohoussou**	Succès 80%	Succès 43%	Non identifié	Non individualisé	Succes72,5%
**Class K Waaldjik**		ICUP 30%			

## Conclusion

La fistule urogénitale reste un réel problème de santé publique. La fistulorraphie en deux plans est certes une technique simple et reproductible mais, l'étroitesse du champ, l'état du tissu local et une lésion possible du sphincter strié rendent difficile l'atteinte des objectifs thérapeutiques. La lutte contre ce fléau nécessite la promotion et l'éducation des filles; la sensibilisation contre les pratiques religieuses et traditionnelles néfastes; le rapprochement des structures fournissant les soins obstétricaux néonataux d'urgences. A ce jour, la mise au point d'un système de classification standard demeure nécessaire, afin qu'on puisse à travers une bonne codification, faciliter la formation, l'évaluation des chirurgiens et la prise de décision thérapeutique.

### Etat des connaissances actuelles sur le sujet

- Contrairement aux pays occidentaux, la fistule d'origine obstétricale est celle qui est prédominante dans notre contexte;
- Pathologie grave à cause de la souffrance physique endurée par les victimes, mais aussi parce qu'elle engendre des conséquences psychosociales et économiques néfastes;
- Parallèlement à l'évolution des techniques chirurgicales, nous avons assisté pendant ces cinquante dernières années, à une multiplication des systèmes de classifications pronostiques, sans qu'aucune ne fasse l'unanimité.

### Contribution de notre étude à la connaissance

- Contrairement aux autres, nous enregistrons un taux de divorce/abandon domicile conjugal assez bas; mais, cela n'est autre que la face visible de l'iceberg car ces bonnes dames sont obligées de renoncer à leur devoir conjugal à cause de l'indifférence et du mépris des proches;
- Cette étude a prouvé qu'un intérêt particulier doit être accordé aux fistules trigono-cervico-utero-vaginale ou la fistule type III du CHU-Point G; elle diffère des autres en termes de voie d'abord, de durée d'intervention et de morbidité per-opératoire; cette entité a été omise dans la classification de Kees Waaldjik et camouflée dans les fistules typeI de Benchekroune;
- Les résultats de cette étude prouvent que la mise au point d'un système de classification standard gage d'une bonne codification et d'une comparaison objective des résultats, est belle et bien possible.

## Conflits des intérêts

Les auteurs ne déclarent aucun conflit d’intérêts.
